# Supplementary material for: Stacking Model‐Based Classifiers for Dealing With Multiple Sets of Noisy Labels
Source: Biom J. 2025 Mar 12;67(2):e70042. doi: 10.1002/bimj.70042 (PMC11898607; doi:10.1002/bimj.70042)
Supplement: Supplementary file 1 — Supporting Information [file BIMJ-67-e70042-s001.zip › density-based-ensemble-model-main/competitors_real_data/netlab-master/netlab-master/help/rbffwd.htm]

Netlab Reference Manual rbffwd


# rbffwd

## Purpose

Forward propagation through RBF network with linear outputs.

## Synopsis

```
a = rbffwd(net, x)
function [a, z, n2] = rbffwd(net, x)
```

## Description

`a = rbffwd(net, x)` takes a network data structure
`net` and a matrix `x` of input
vectors and forward propagates the inputs through the network to generate
a matrix `a` of output vectors. Each row of `x` corresponds to one
input vector and each row of `a` contains the corresponding output vector.
The activation function that is used is determined by `net.actfn`.

`[a, z, n2] = rbffwd(net, x)` also generates a matrix `z` of
the hidden unit activations where each row corresponds to one pattern.
These hidden unit activations represent the `design matrix` for
the RBF. The matrix `n2` is the squared distances between each
basis function centre and each pattern in which each row corresponds
to a data point.

## Examples

```
[a, z] = rbffwd(net, x);

temp = pinv([z ones(size(x, 1), 1)]) * t;
net.w2 = temp(1: nd(2), :);
net.b2 = temp(size(x, nd(2)) + 1, :);
```

Here `x` is the input data, `t` are the target values, and we use the
pseudo-inverse to find the output weights and biases.

## See Also

`rbf`, `rbferr`, `rbfgrad`, `rbfpak`, `rbftrain`, `rbfunpak`

---

**Pages:**
Index


---

Copyright (c) Ian T Nabney (1996-9)
